# Effects of mixed blood metals on hearing loss among steel workers: a case-control study

**DOI:** 10.3389/fpubh.2026.1863565

**Published:** 2026-07-07

**Authors:** Jian Zhang, Junwei He, Yifan Zhang, Mengshi Chen, Jing Deng

**Affiliations:** Department of Epidemiology and Health Statistics, Xiangya School of Public Health, Central South University, Changsha, Hunan, China

**Keywords:** Bayesian kernel machine regression, hearing loss, mixed metal exposure, noise, steel workers, weighted quantile sum

## Abstract

**Objectives:**

To investigate the associations of single and multiple blood metal exposures with hearing loss, and evaluate the interactions between metals and noise exposure, thereby providing scientific evidence for the prevention and control of hearing loss in occupational populations.

**Methods:**

This study investigates the associations between 17 blood metals and hearing loss among steel workers. A matched case-control study was conducted, including 300 cases with hearing loss and 300 age- and sex-matched controls, selected from 4,081 eligible workers. Metal concentrations were measured by inductively coupled plasma mass spectrometry (ICP-MS). Associations were estimated by multivariable logistic regression, LASSO regression, weighted quantile sum (WQS) regression, and Bayesian kernel machine regression (BKMR). The most important metals were identified, and their additive interactions with noise exposure were further assessed.

**Results:**

Cases and controls were comparable in age, sex, and work tenure (all *P* > 0.05). Blood levels of Mn, Cu, As, Se, and Sr were significantly higher in cases than in controls (all *P* < 0.05). Logistic regression showed that Mn (OR = 4.68, 95%CI: 2.88–7.59), Cu (OR = 3.30, 1.55–7.04), As (OR = 1.14, 1.05–1.22), Se (OR = 4.25, 2.16–8.37), and Sr (OR = 8.22, 4.56–14.80) were associated with increased hearing loss risk. WQS analysis indicated a positive association between metal mixture and hearing loss (OR = 6.52, 95%CI: 3.61–11.78), with Se (0.261) and Sr (0.256) having the highest weights. BKMR further identified Mn and Sr as the most important predictors. Significant additive interactions were found respectively for Mn and Sr with noise exposure: RERI = 11.36 (5.72–39.38) and 17.53 (8.07–66.64).

**Conclusion:**

Noise and multiple metals (particularly Mn and Sr) were significantly associated with hearing loss in steel workers, with potential additive interactions observed between these exposures. Therefore, comprehensive occupational health strategies addressing both noise and metal exposures may be important for hearing loss prevention.

## 1 Introduction

The World Health Organization (WHO) estimates that approximately 430 million people globally are affected by disabling hearing loss, with occupational noise-induced hearing loss (NIHL) being the most prevalent and irreversible form of hearing impairment (1). NIHL is recognized as one of the most common and irreversible occupational diseases worldwide. Its pathogenesis is primarily attributed to both mechanical and metabolic damage to the cochlear hair cells and synaptic structures resulting from prolonged or high-intensity noise exposure. Initial manifestations of NIHL include high-frequency hearing loss, which gradually progresses to speech-frequency hearing impairment, severely impacting workers' communication abilities, quality of life, and mental health (2, 3). In the United States, around 22 million workers are exposed to noise levels that have the potential to damage hearing, while in China, more than 80% of industrial enterprises face noise hazards, with approximately 80 million workers subjected to harmful noise level (4, 5). Despite ongoing efforts to strengthen prevention and control measures globally, the burden of NIHL continues to rise, particularly in rapidly industrializing middle-income countries (6, 7).

The steel industry is considered a high-risk sector for noise-induced hearing loss (NIHL), with its prevalence among steelworkers typically ranging from 20 to 25%, which is significantly higher than that observed in the general workforce or in workers employed in low-noise industries (approximately 5%−15%) (4, 8, 9). Numerous cross-sectional studies conducted both domestically and internationally have consistently demonstrated that prolonged noise exposure is an independent risk factor for hearing loss among workers. For instance, a study conducted in the Egyptian steel industry revealed that the risk of NIHL among workers exposed to noise was 6.55 times greater than that of unexposed workers (10–12).

Within the high-temperature chemical reaction environment characteristic of steel production, noise is not the sole ototoxic hazard. The manufacturing process generates substantial quantities of metal fumes and particulate matter enriched with heavy metals, including lead, manganese, cadmium, chromium, and arsenic (13). Lead and cadmium primarily originate from scrap steel coatings and ore impurities; during high-temperature smelting, these elements volatilize and form oxide aerosols. Upon inhalation, such aerosols may induce both neurotoxic and ototoxic effects in exposed workers (14–16). In addition, metals such as manganese, chromium, and nickel are released in considerable quantities during welding and smelting operations (17, 18). Existing evidence has identified lead and cadmium as major metallic risk factors for hearing impairment (19, 20). Moreover, the synergistic interaction between occupational noise exposure and heavy metal exposure substantially increases the risk of hearing loss. Hwang et al. (21)reported a significant interaction between blood lead levels and noise exposure among steelworkers, with a markedly higher risk of hearing loss observed in workers co-exposed to high lead levels and high noise compared with those exposed to high noise alone. This finding was further corroborated by Choi et al. (22). Additionally, Chen et al. (23)demonstrated that the combined effects of lead exposure and noise may potentiate the risk of high-frequency auditory damage.

Although previous studies have investigated the otological effects of heavy metal exposure—either alone or in combination with noise—among steelworkers, these investigations have predominantly focused on lead and cadmium. Research on other metals, such as manganese, arsenic, and chromium, remains limited and is largely confined to animal models (24, 25). Furthermore, the current understanding of the combined effects of mixed heavy metal exposures—particularly those involving metals other than lead and cadmium—together with noise exposure on auditory function remains incomplete.

Hunan Province is located at the intersection of the mineral-rich belt in the middle and lower reaches of the Yangtze River and a major polymetallic ore deposit region, resulting in a widespread distribution of heavy metals. Concurrently, the steel industry represents a key pillar of the local economy in this region. To date, no studies have systematically examined the association between heavy metal exposure and hearing loss among steelworkers in Hunan Province. The present study aims to evaluate the combined associations between heavy metal exposure and occupational noise exposure by measuring blood concentrations of multiple heavy metals in steelworkers and integrating these data with noise exposure assessment records. This approach is expected to elucidate both the independent and synergistic effects of heavy metals in the development of noise-induced hearing loss. Furthermore, the findings will provide more comprehensive epidemiological evidence to inform the development of effective hearing conservation and occupational health protection strategies for steelworkers, thereby supporting efforts toward prevention and risk reduction in occupational health.

## 2 Materials and methods

### 2.1 Research design and subjects

This study employed a case-control design, utilizing data collected by our research team from January to December 2021. The data pertain to active employees of Xiangtan Iron and Steel Group who underwent occupational health examinations at Xiangtan Central Hospital. The inclusion criteria were as follows: (1) age 18 years or older; (2) normal cognitive function and the ability to communicate effectively; (3) occupation requiring occupational health examinations in accordance with the “Technical Specifications for Occupational Health Surveillance” (GBZ188-2014); (4) voluntary participation in the study with written informed consent. The exclusion criteria included: (1) incomplete examination data; (2) a history of ear diseases such as otitis media, acoustic neuroma, hearing impairment, hearing loss, deafness, or occupational noise-induced hearing loss. A total of 4,081 steelworkers met the eligibility criteria, of whom 664 exhibited abnormal hearing. For this study, 300 workers with abnormal hearing were randomly selected as the case group, and 300 workers with normal hearing were selected as the control group through frequency-matched by gender and age, resulting in a total of 600 participants.

### 2.2 Data collection

#### 2.2.1 Collection of basic information

Participants in the study underwent the following surveys and examinations: (1) Questionnaire survey, which included sociodemographic characteristics (sex, age, marital status, education, occupation, etc.), lifestyle factors (smoking, alcohol consumption, physical activity, etc.), dietary habits (consumption of smoked foods, pickled foods, tea, etc.), and work conditions (exposure to high temperatures, noise, and other occupational hazards). (2) Physical examination, which involved basic measurements, including weight (kg), height (m), waist circumference (WC), and blood pressure. Blood pressure was measured three times for each participant using an electronic sphygmomanometer (Omron; Dalian, China) after 10 min of rest. The blood pressure value recorded in this study was the average of these three measurements. Participants were instructed to fast for at least 10 h before providing blood samples the following morning. (3) Laboratory tests, including fasting blood glucose (FBG), total cholesterol (TC), triglycerides (TG), low-density lipoprotein cholesterol (LDL-C), high-density lipoprotein cholesterol (HDL-C), serum uric acid (SUA), and creatinine. All questionnaires were administered by uniformly trained nurses, and all physical examinations were conducted by qualified physicians at the Health Management Center of Xiangtan Central Hospital according to standard procedures. Laboratory tests were carried out at the central laboratory of Xiangtan Central Hospital (Hunan, China).

#### 2.2.2 Hearing test

Pure-tone air-conduction threshold tests were conducted on all study participants using an AS216 audiometer (Interacoustics AS, Denmark), which was calibrated prior to use. Testing was carried out in a soundproof room with a background noise level of <25 dB(A). The tests were performed at frequencies of 500, 1,000, 2,000, 3,000, 4,000, and 6,000 Hz. Test results were adjusted for gender and age in accordance with GB/T 7,582, “Statistical Distribution of Hearing Thresholds in Relation to Age.

#### 2.2.3 Heavy metal testing

At the health examination site, nurses in the phlebotomy room collected fasting venous blood from each participant using ethylenediaminetetraacetic acid (EDTA-K2) anticoagulant tubes. After centrifugation, the samples were stored in a −80 °C freezer for later analysis. Heavy metal testing was conducted at the Experimental Center of the Xiangya School of Public Health, Central South University. Concentrations of 17 metal elements in the participants' blood were measured using an inductively coupled plasma mass spectrometer (ICP-MS, Agilent Technologies 7,900). The elements measured included titanium (Ti), vanadium (V), chromium (Cr), manganese (Mn), iron (Fe), cobalt (Co), nickel (Ni), copper (Cu), zinc (Zn), arsenic (As), selenium (Se), strontium (Sr), cadmium (Cd), antimony (Sb), barium (Ba), lead (Pb), and molybdenum (Mo).

### 2.3 Related variables and definitions

#### 2.3.1 Definition of hearing loss

Hearing loss: According to the Chinese Diagnostic Criteria of Occupational Noise-Induced Deafness (GBZ 49-2014), individuals diagnosed with any of the above types of hearing loss are defined as having hearing loss. (1) Speech-frequency hearing loss: An average hearing threshold of >25 dB at 500 Hz, 1,000 Hz, and 2,000 Hz in both ears. (2) High-frequency hearing loss: An average hearing threshold of ≥40 dB at 3,000 Hz, 4,000 Hz, and 6,000 Hz in both ears. (3) Suspected noise-induced hearing loss: Individuals with an average hearing threshold of ≥40 dB at 3,000 Hz, 4,000 Hz, and 6,000 Hz in both ears, and a weighted average of ≥26 dB at 500 Hz, 1,000 Hz, 2,000 Hz, and 4,000 Hz in the better ear.

#### 2.3.2 Other variables and their definitions

(1) Hypertension: In accordance with the *Chinese Guidelines for the Prevention and Treatment of Hypertension (2024 Revised Edition)*, the diagnostic criteria for hypertension in this study are as follows: Hypertension is defined by meeting any of the following conditions: (1) having been informed by a physician or healthcare professional that the individual has hypertension; (2) having a systolic blood pressure (SBP) ≥140 mmHg or diastolic blood pressure (DBP) ≥90 mmHg on three separate occasions; (3) currently taking antihypertensive medication.

(2) Diabetes: In accordance with the *Chinese Guidelines for the Prevention and Treatment of Type 2 Diabetes (2020 Edition)*, normal blood glucose is defined as fasting plasma glucose (FPG) <6.1 mmol/L. Diabetes is defined by meeting any of the following criteria: (1) FPG ≥6.1 mmol/L; (2) a medical diagnosis of impaired fasting glucose or diabetes; (3) a history of diabetes.

(3) Dyslipidemia: The clinical classification criteria for dyslipidemia, as outlined in the *Chinese Guidelines for the Prevention and Treatment of Dyslipidemia in Adults (2016 Revised Edition)*, were used in this study. Dyslipidemia is defined by meeting any of the following criteria: (1) hypercholesterolemia: total cholesterol (TC) ≥6.2 mmol/L; (2) hypertriglyceridemia: triglycerides (TG) ≥2.3 mmol/L; (3) hyper-low-density lipoprotein cholesterol (hyper-LDL-C): low-density lipoprotein cholesterol (LDL-C) ≥4.1 mmol/L; (4) low high-density lipoprotein cholesterol (low HDL-C): HDL-C <1.0 mmol/L; (5) a history of dyslipidemia.

(4) Smoking: Smoking status in this study was categorized based on participants' responses to questionnaire items regarding ”whether they smoke,“ ”number of years smoked,“ ”number of cigarettes smoked per day,“ and ”number of years since quitting.“ Smoking status was classified into three groups: current smokers, former smokers (those who no longer smoke), and never-smokers.

(5) Alcohol consumption: Based on the definition of alcohol consumption provided by the Chinese Center for Disease Control and Prevention and participants' responses to the questionnaire regarding ”whether they drink alcohol,“ ”type of alcohol consumed,“ ”number of years of drinking,“ and ”frequency of drinking," alcohol consumption was classified into three groups: current drinkers, former drinkers (those who no longer drink), and never-drinkers.

(6) Definition of Occupational Hazard Exposure: Occupational hazard exposure was determined based on the specific roles and job types of employees in steel enterprises. The Occupational Disease Prevention and Control Institute assesses exposure to hazardous factors for each position by analyzing historical hazard monitoring data and conducting periodic inspections. In this study, occupational hazards are defined as ”hazard factors‘' identified in employees' physical examination reports, primarily including noise, high temperatures, carbon monoxide, and dust.

### 2.4 Statistical analysis

In the comparison of baseline characteristics between cases and controls, continuous variables that followed a normal distribution were expressed as mean ± standard deviation (X ± S) and analyzed using the *t*-test. Continuous variables that did not follow a normal distribution were expressed as the median and interquartile range (M(QR)) and analyzed using the Mann-Whitney U test. Categorical data were presented as the number of cases (*n*) and proportion (*n* (%)), and analyzed using the chi-square (χ^2^) test. Since plasma metal concentrations exhibited a right-skewed distribution, all subsequent analyses were performed after log-transforming the data. Spearman's rank correlation analysis was used to assess correlations between blood metal concentrations in the case-control cohort. Based on frequency-matched design, unconditional logistic regression was employed to investigate the association between single heavy metal exposure and hearing loss, while the least absolute shrinkage and selection operator (LASSO) regression method was used to identify key metals associated with hearing loss. A weighted quintile sum (WQS) regression model was used to analyze the association of combined heavy metal exposure and the relative contributions of each metal. A WQS index of *P* < 0.05 was used to determine the association between mixed exposure and hearing loss, with higher weights indicating greater contributions. A Bayesian kernel machine regression (BKMR) model was employed to further validate the relationship between mixed exposure and hearing loss. This model is suitable for analyzing exposure-response relationships between complex environmental mixtures and health outcomes, as it can assess both single-exposure effects and interactions between exposures. Element importance was determined using posterior inclusion probability (PIP); values closer to 1 indicate higher importance, while values closer to 0 indicate lower importance. Metals with PIP values close to 1 in the BKMR model were divided into high- and low-exposure groups based on the median. For WQS and BKMR analyses, age, sex, and other potential confounders were included as covariates. These groups were combined with the noise analysis to assess additive interactions, calculating the Relative Excess Risk due to Interaction (RERI), the Attributable Proportion due to Interaction (AP), and the Synergy Index (S) along with their 95% confidence intervals (CIs). All statistical analyses were performed using SPSS 27.0 and R 4.4.2 software.

## 3 Results

### 3.1 Basic characteristics of the study population

This study included 600 participants, with 300 in the control group and 300 in the case group. In the case group, there were 277 males (92.3%) and 23 females (7.7%); in the control group, there were 278 males (92.6%) and 22 females (7.3%). No statistically significant difference in gender distribution was observed between the case and control groups (*P* = 0.907). In the case group, 5 participants (1.7%) were under 30 years of age, 150 (50.0%) were aged 30–49, and 145 (48.3%) were aged 50 or older; the control group included 4 individuals (1.3%) under 30 years, 154 (51.3%) aged 30–49, and 142 (47.3%) aged 50 or older. No statistically significant difference in age distribution was found between the two groups (*P* = 0.169). In the case group, 46 participants (15.3%) had less than 10 years of service, 82 (27.3%) had 10–19 years, 69 (23.0%) had 20–29 years, 101 (33.7%) had 30–39 years, and 2 (0.7%) had 40 or more years of service. In the control group, 44 participants (14.7%) had less than 10 years of service, 64 (21.3%) had 10–19 years, 76 (25.3%) had 20–29 years, 108 (36.0%) had 30–39 years, and 8 (2.7%) had 40 or more years of service. No statistically significant difference in years of service distribution was found between the case and control groups (*P* = 0.169). Chi-square test results revealed statistically significant differences between the case and control groups in terms of educational level, noise exposure, heat exposure, dust exposure, earplug use, and mask use (*P* < 0.05). However, no statistically significant differences were found in marital status, hypertension, diabetes, dyslipidemia, body mass index (BMI), smoking, or alcohol consumption. Details are provided in Table 1.

**Table 1:** Baseline characteristics of the case and control group.

Variable	group	Control (*n* = 300)	Case (*n* = 300)	*P*-values
Age, *n* (%)	<30 years	4 (1.3)	5 (1.7)	0.907
	0-49 years	154 (51.3)	150 (50.0)	
	≥50 years	142 (47.3)	145 (48.3)	
Work tenure, *n* (%)	<10 years	44 (14.7)	46 (15.3)	0.169
	10–19 years	64 (21.3)	82 (27.3)	
	20–29 years	76 (25.3)	69 (23.0)	
	30–39 years	108 (36.0)	101 (33.7)	
	≥40 years	8 (2.7)	2 (0.7)	
Gender, *n* (%)	Male	278 (92.6)	277 (92.3)	0.877
	Female	22 (7.3)	23 (7.7)	
Education, *n* (%)	Middle school or below	85 (28.3)	108 (36.0)	**0.042**
	High school/technical school	143 (47.7)	141 (47.0)	
	College degree or above	72 (24.0)	51 (17.0)	
Marital status, *n* (%)	Unmarried	11 (3.7)	13 (4.3)	0.917
	Married	266 (88.7)	264 (88.0)	
	Divorced/widowed	23 (7.7)	23 (7.7)	
Noise exposure, *n* (%)	No	164 (54.7)	51 (17.0)	**<0.001**
	Yes	136 (45.3)	249 (83.0)	
High temperature exposure, *n* (%)	No	245 (81.7)	265 (88.3)	**0.022**
	Yes	55 (18.3)	35 (11.7)	
Dust exposure, *n* (%)	No	197 (65.7)	230 (76.7)	**0.003**
	Yes	103 (34.3)	70 (23.3)	
CO exposure, *n* (%)	No	266 (88.7)	279 (93.0)	0.066
	Yes	34 (11.3)	21 (7.0)	
Earplug use, *n* (%)	No	170 (56.7)	67 (22.3)	**<0.001**
	Yes	130 (43.3)	233 (77.7)	
Mask use, *n* (%)	No	202 (67.3)	233 (77.7)	**0.005**
	Yes	98 (32.7)	67 (22.3)	
Hypertension, *n* (%)	No	219 (73.0)	206 (68.7)	0.243
	Yes	81 (27.0)	94 (31.3)	
Diabetes, *n* (%)	No	235 (78.3)	229 (76.3)	0.559
	Yes	65 (21.7)	71 (23.7)	
Dyslipidemia, *n* (%)	No	150 (50.0)	169 (56.3)	0.120
	Yes	150 (50.0)	131 (43.7)	
BMI, *n* (%)	<24	130 (43.3)	128 (42.7)	0.841
	24~	119 (39.7)	127 (42.3)	
	28~	51 (17.0)	45 (15.0)	
Smoking, *n* (%)	Current	187 (62.3)	192 (64.0)	0.894
	Former	32 (10.7)	32 (10.7)	
	Never	81 (27.0)	76 (25.3)
Alcohol consumptiol, *n* (%)	Current	122 (40.7)	120 (40.0)	0.719
	Former	62 (20.7)	70 (23.3)	
	Never	116 (38.7)	110 (36.7)	

Bold values indicate statistical significance (*P* < 0.05).

### 3.2 Distribution of blood metal concentrations in the study population

A total of 17 metals were analyzed in this study, and all their concentrations exhibited a right-skewed distribution. The concentrations of blood metals Mn, Cu, As, Se, and Sr in the case group were significantly higher than those in the control group, while the concentration of Ti in the case group was significantly lower than that in the control group. Details are provided in Table 2.

**Table 2:** Distribution of blood metal concentrations in the study population.

Metal	Total (μg/L)	Control (μg/L)	Case (μg/L)	*P*-value
Ti	93.51 (66.00, 116.44)	96.08 (64.01, 123.56)	90.55 (67.11, 111.05)	**0.046**
V	1.32 (0.65, 2.30)	1.23 (0.66, 2.07)	1.46 (0.63, 2.44)	0.115
Cr	83.17 (56.31, 119.80)	82.04 (57.70, 115.53)	84.62 (55.52, 128.87)	0.421
Mn	34.50 (25.01, 44.52)	31.35 (21.70, 41.34)	37.80 (29.58, 47.74)	**<0.001**
Fe^*^	199.88 (657.95, 258.18)	180.76 (48.13, 255.56)	213.14 (78.85, 266.24)	0.051
Co	1.00 (0.27, 2.13)	0.77 (0.13, 2.53)	1.10 (0.50, 1.87)	0.400
Ni	9.88 (6.21, 18.85)	11.74 (6.61, 19.27)	8.91 (5.85, 18.35)	0.062
Cu	736.37 (632.61, 847.68)	723.88 (612.60, 820.97)	750.52 (639.65, 868.14)	**0.008**
Zn^*^	7.92 (5.70, 10.05)	7.64 (5.51, 10.17)	8.05 (6.07, 9.89)	0.638
As	1.07 (0.01, 1.77)	0.45 (0.01, 1.97)	1.286 (0.27, 1.72)	**0.004**
Se	87.40 (74.88, 105.27)	83.75 (70.94, 100.31)	90.38 (79.32, 108.74)	**<0.001**
Sr	47.82 (36.92, 60.53)	43.47 (33.68, 55.27)	53.72 (42.78, 67.99)	**<0.001**
Mo	22.25 (7.80, 39.23)	20.12 (8.03, 37.92)	24.20 (7.90, 42.53)	0.345
Cd	0.79 (0.07, 3.56)	0.618 (0.06, 3.88)	1.106 (0.12, 3.39)	0.814
Sb	37.57 (22.73, 64.96)	34.64 (21.28, 70.49)	39.83 (25.62, 63.51)	0.351
Ba	12.31 (2.86, 37.37)	19.26 (1.52, 39.27)	8.53 (3.49, 32.30)	0.279
Pb	24.69 (16.43, 35.03)	24.31 (15.01, 37.56)	24.85 (17.19, 32.59)	0.809

Blood metal concentrations showed a skewed distribution, thus presented as median (interquartile range; Non-parametric tests were used. ^*^Units are mg/L for Fe and Zn; all other metals are in μg/L. Bold values indicate statistical significance (*P* < 0.05).

### 3.3 Correlation analysis of plasma metals

**Figure 1:**
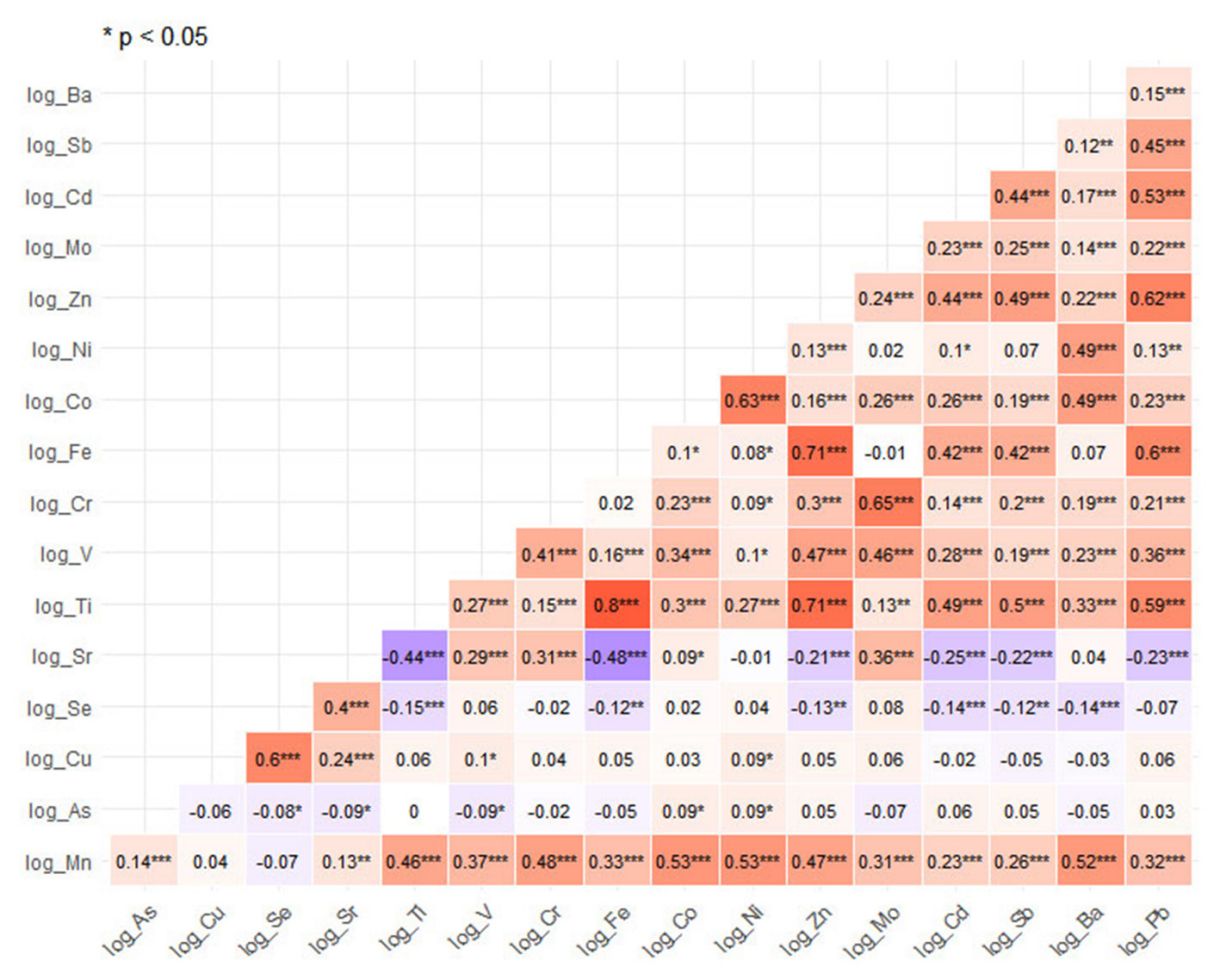
Analysis of metals in blood. *P < 0.05, **P < 0.01, ***P < 0.001.

The correlations among the metals are shown in Figure 1. Most metals exhibited significant correlations, with Spearman's correlation coefficients ranging from −0.48 to 0.80. The strongest positive correlation was observed between Fe and Ti (*r* = 0.80, *P* < 0.001), while the strongest negative correlation was observed between Fe and Sr (*r* = −0.48, *P* < 0.001).

### 3.4 Analysis of the association between exposure to individual heavy metals and hearing loss

Logistic regression was used to analyze the association between exposure to individual heavy metals and hearing loss. Metal concentrations were log-transformed before being included in the logistic regression model. After adjusting for relevant factors such as gender and years of service, Mn (OR = 4.677, 95% CI: 2.883–7.588), Cu (OR = 3.298, 95% CI: 1.546–7.035), As (OR = 1.136, 95% CI: 1.054–1.224), Se (OR = 4.251, 95% CI: 2.160–8.369), and Sr (OR = 8.215, 95% CI: 4.560–14.800) were significantly associated with an increased risk of hearing loss. Detailed results for both adjusted and unadjusted analyses are presented in Table 3.

**Table 3:** Association between single metal exposure and hearing loss.

Variable	Model 1^*^		Model 2^**^	
	OR(95%CI)	*P*-value	OR(95%CI)	*P*-value
Ti	0.769 (0.513,1.152)	0.203	0.699 (0.439,1.112)	0.131
V	0.991 (0.917,1.071)	0.821	1.032 (0.944,1.127)	0.491
Cr	1.101 (0.842,1.440)	0.481	1.211 (0.892,1.642)	0.219
Mn	4.287 (2.806,6.549)	**<0.001**	4.677 (2.883,7.588)	**<0.001**
Fe^*^	1.120 (0.987,1.270)	0.079	1.082 (0.933,1.254)	0.298
Co	1.021 (0.938,1.112)	0.627	1.036 (0.941,1.140)	0.477
Ni	0.792 (0.655,0.958)	**0.016**	0.877 (0.712,1.081)	0.218
Cu	2.494 (1.266,4.916)	**0.008**	3.298 (1.546,7.035)	**0.002**
Zn^*^	1.109 (0.770,1.598)	0.578	0.868 (0.683,1.572)	0.868
As	1.164 (1.090,1.242)	**<0.001**	1.136 (1.054,1.224)	**<0.001**
Se	3.326 (1.817,6.088)	**<0.001**	4.251 (2.160,8.369)	**<0.001**
Sr	5.870 (3.539,9.737)	**<0.001**	8.215 (4.560,14.800)	**<0.001**
Mo	0.968 (0.895,1.047)	0.412	0.982 (0.898,1.074)	0.696
Cd	0.987 (0.926,1.052)	0.680	0.982 (0.912,1.056)	0.621
Sb	0.995 (0.793,1.247)	0.963	1.015 (0.784,1.313)	0.912
Ba	1.004 (0.930,1083)	0.928	1.016 (0.932,1.108)	0.719
Pb	1.021 (0.794,1.311)	0.874	1.079 (0.807,1.441)	0.608

^*^Model 1: unadjusted;^**^Model 2: adjusted for gender, work tenure, noise, high temperature, dust, CO, hypertension, diabetes, dyslipidemia, BMI, education, marital status, alcohol, smoking, earplug use, mask use. Bold values indicate statistical significance (*P* < 0.05).

### 3.5 Association between mixed metal exposure and hearing loss

#### 3.5.1 LASSO regression to identify metal factors with a primary effect on occupational noise-induced hearing loss

**Figure 2:**
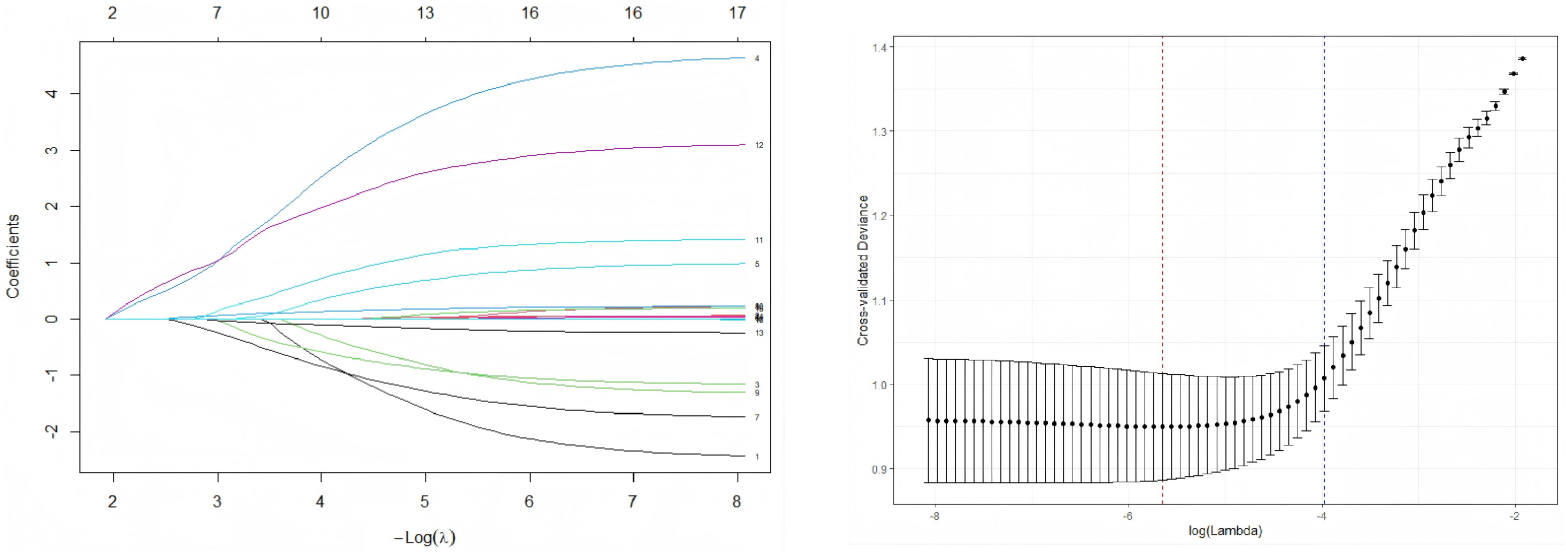
Results of LASSO model screening of main effect metals.

A LASSO regression model was used to screen 17 metal elements to identify those with the most significant influence on hearing loss. During the analysis, all 17 metals were included, with adjustments made for covariates. The results show the distribution of the mean squared error and all λ values. The metals included in the model at the optimal λ (corresponding to the minimum mean squared error) were selected as the final results (Figure 2). A total of 10 major metal elements were ultimately identified: titanium, arsenic, molybdenum, zinc, manganese, chromium, selenium, strontium, antimony, and iron.

#### 3.5.2 WQS model analysis of the association between mixed metal exposure and hearing loss

**Figure 3:**
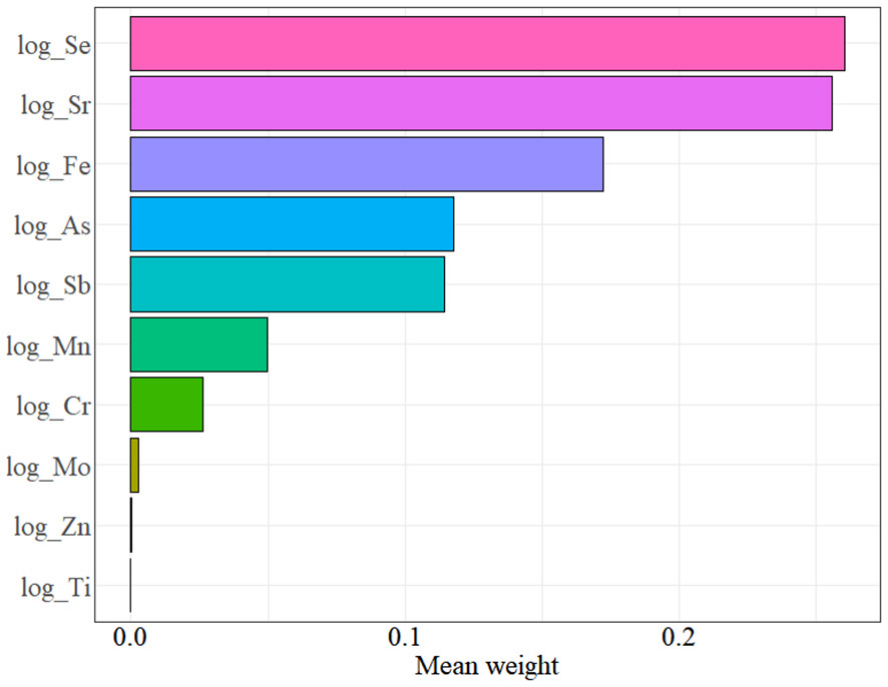
Weights of each metal element in the WQS model. The model adjusted for gender, years of service, noise exposure, high temperature exposure, dust exposure, CO exposure, hypertension, diabetes, dyslipidemia, BMI, educational level, marital status, alcohol consumption, smoking, earplug use, and mask use.

The WQS regression model assumes that all exposure variables are associated with the outcome in the same direction, so both positive and negative directional models were evaluated in this study. The results showed that, in the positive-direction model (Figure 3), the WQS index for blood metal mixture exposure was significantly associated with hearing loss risk (OR = 6.52, 95% CI: 3.61–11.78). Among the included metals, Se (0.261) and Sr (0.256) contributed the highest weights, followed by Fe (0.172), As (0.118), and Sb (0.114). In contrast, the negative-direction model showed no statistically significant association between the WQS index and hearing loss risk (OR = 1.01, 95% CI: 0.72–1.41).

#### 3.5.3 Analysis of the association between mixed metal exposure and hearing loss using the BKMR model

In the BKMR analysis, the 10 metals were grouped based on inter-metal correlation structures, occupational exposure characteristics, and potential biological relevance. Specifically, As, Mn, Cr, Sb, and Se were classified into one group, while Fe, Zn, Sr, Ti, and Mo were classified into another group. This grouping strategy was applied to better reflect the correlated structure of mixed metal exposures in the steelworking environment and to improve model stability in the BKMR analysis, following grouping approaches commonly used in studies of correlated environmental mixtures. Table 4 presents the posterior inclusion probabilities (PIPs) for each metal, with Mn (1.000) and Sr (0.926) identified as the most influential predictors in the final BKMR model.

**Table 4:** Posterior inclusion probabilities (PIP) from BKMR model.

Variable	Group	Group PIP	Conditional PIP
log_Ti	1	1	0.074
log_Mn	2	1	1.000
log_As	2	1	0.000
log_Fe	1	1	0.000
log_Se	2	1	0.000
log_Sr	1	1	0.926
log_Zn	1	1	0.000
log_Sb	2	1	0.000
log_Mo	1	1	0.000
log_Cr	2	1	0.000

Metal concentrations were log-transformed before entering the logistic regression; PIP close to 1 indicates high importance.

**Figure 4:**
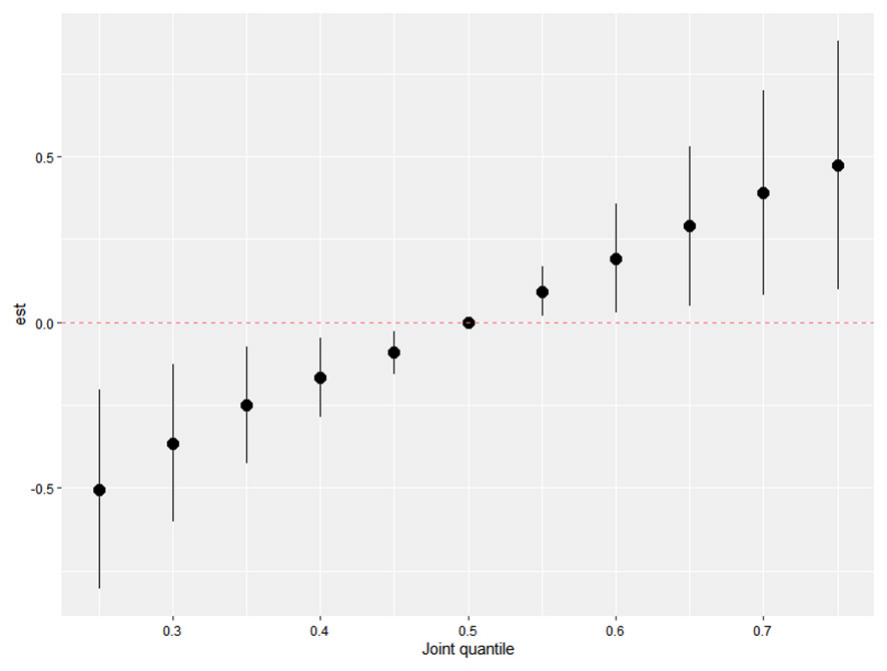
Association between BKMR model joint exposure and hearing loss.

**Figure 5:**
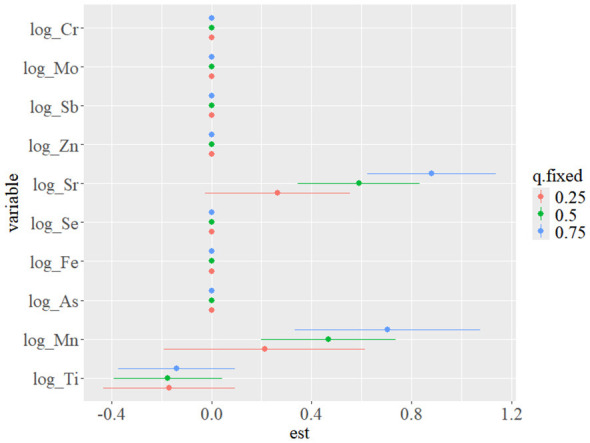
Single exposure effect.

**Figure 6:**
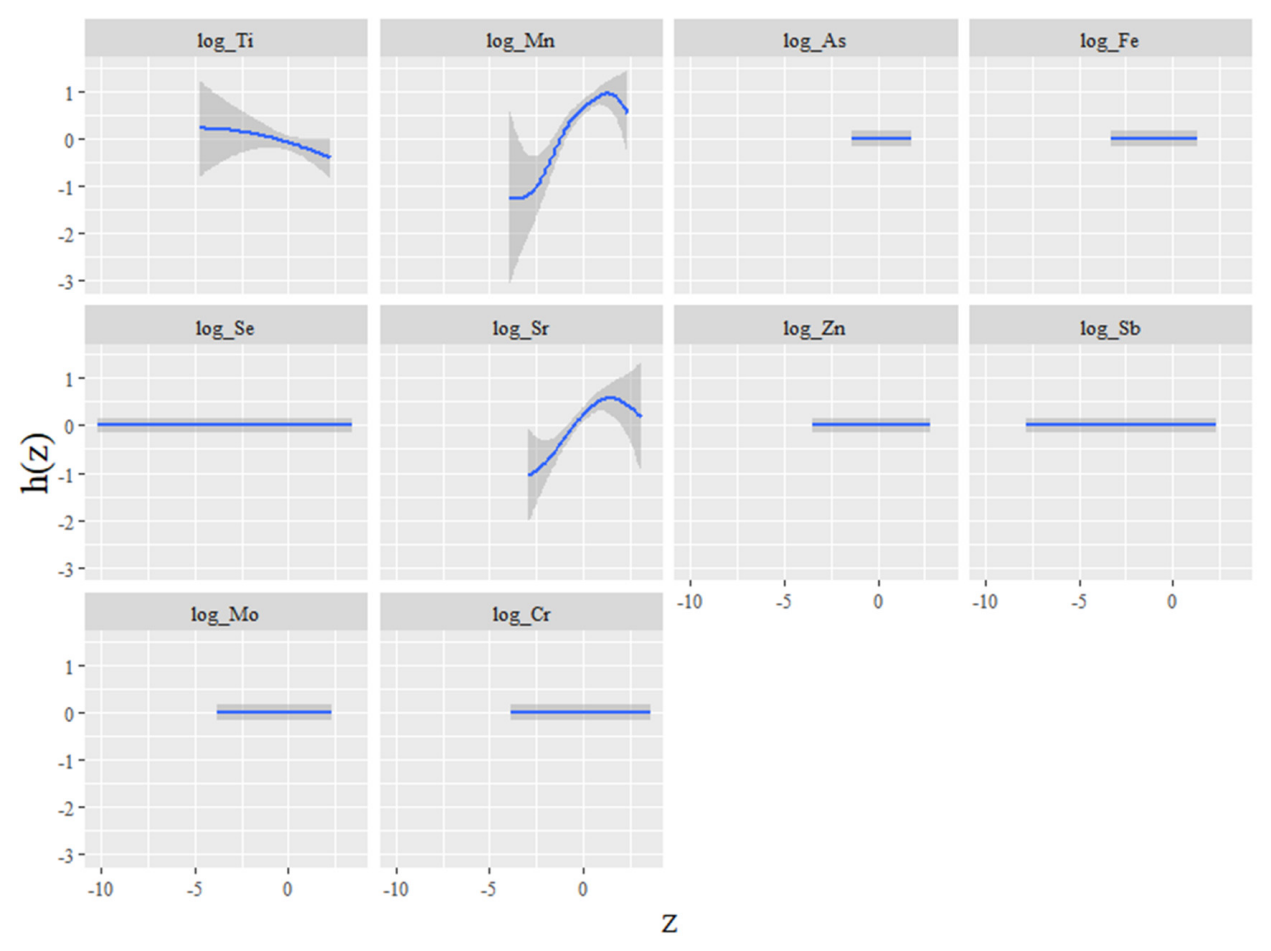
Single exposure dose-response relationship.

**Figure 7:**
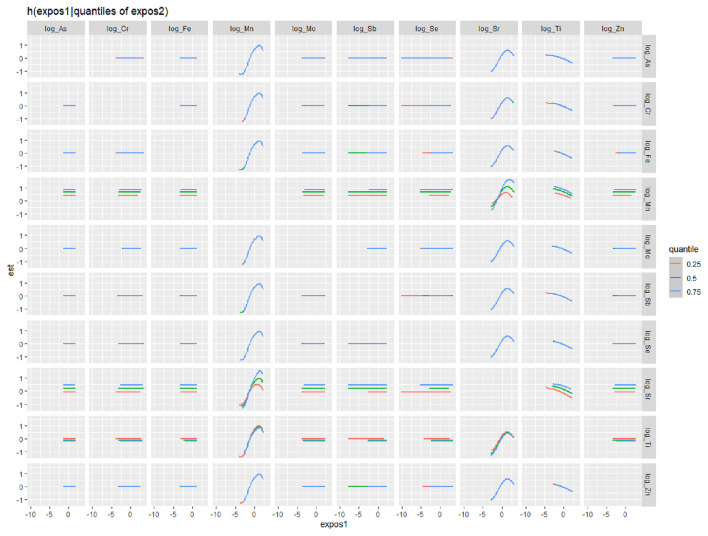
Interactions of mixed exposure.

As shown in Figure 4, blood metal mixture exposure was positively associated with hearing loss, with higher mixture concentrations corresponding to an increased risk of hearing loss. We further evaluated the effect estimates of individual metals within the mixture by examining the association between each single metal and hearing loss while fixing the concentrations of the remaining metals at the P25, P50, and P75 levels. As shown in Figure 5, Mn and Sr demonstrated significant positive associations with hearing loss, whereas the associations for Fe, Cr, Mo, Sb, Zn, Se, As, and Ti were not statistically significant. The dose–response relationships between individual metals and hearing loss are presented in Figure 6. When the concentrations of all other metals were fixed at the P50 level, Sr and Mn showed significant positive and nonlinear associations with hearing loss, while Fe demonstrated a linear positive association with hearing loss. No obvious associations were observed between hearing loss and Cr, Mo, Sb, Zn, Se, As, or Ti. The interaction analysis from the BKMR model (Figure 7) further suggested a potential positive synergistic interaction between Mn and Sr.

### 3.6 Analysis of the interaction between metals and noise

As shown in Tables 5, 6, the results of the additive interaction analysis which indicate a significant additive interaction between Mn and noise. Compared with the group exposed to low levels of Mn in the absence of noise, the odds ratio (OR) for the group exposed to high levels of Mn in the presence of noise was 18.82 (95% CI: 6.99, 50.70). The ORs and 95% CIs for the Relative Excess Risk due to Interaction (RERI), Attributable Proportion (AP), and Synergy Index (S) were 11.36 (5.72, 39.38), 0.60 (0.32, 0.74), and 2.76 (1.64, 4.63), respectively. A significant additive interaction was also observed between Sr and noise. Compared with the group exposed to no noise and low Sr levels, the OR for the group exposed to noise and high Sr levels was 29.96 (95% CI: 10.56, 85.00). The ORs and 95% CIs for RERI, AP, and S were 17.53 (8.07, 66.64), 0.59 (0.24, 0.72), and 2.53 (1.49, 4.32), respectively. Both models were adjusted for sex, years of work, high temperature, dust, CO exposure, marital status, BMI, smoking, alcohol consumption, hypertension, diabetes, blood lipids, education level, earplug use, and mask use. Additionally, Table 5 was further adjusted for strontium concentration, and Table 6 was further adjusted for manganese (Mn) concentration.

**Table 5:** Interaction between manganese and noise exposure.

Noise exposure	Manganese level	OR(95%CI)
No	Low(ref)	1
	High	1.85(0.95,3.63)
Yes	Low	6.61(2.55,17.10)
	High	18.82(6.99,50.70)
Multiplicative	–	1.54(0.68,3.50)
Additive	RERI	11.36(5.72,39.38)
	AP	0.60(0.32,0.74)
	S	2.76(1.64,4.63)

RERI, relative excess risk due to interaction; AP, attributable proportion due to interaction; S, Synergy Index.

**Table 6:** Interaction between strontium and noise exposure.

Noise exposure	Strontium level	OR(95%CI)
No	Low(ref)	1
	High	4.82(2.31,10.04)
Yes	Low	8.61(3.03,24.46)
	High	29.96(10.56,85.00)
Multiplicative	–	0.72(0.30,1.73)
Additive	RERI	17.53(8.07,66.64)
	AP	0.59(0.24,0.72)
	S	2.53(1.49,4.32)

RERI, relative excess risk due to interaction; AP, attributable proportion due to interaction; S, Synergy Index.

## 4 Discussion

This study found, through various statistical models, that steelworkers exposed to Mn, Cu, As, Se, and Sr—either individually or in combination—had a significantly increased risk of hearing loss. Among these, Mn and Sr were identified as key metals, and a significant additive interaction between Mn, Sr, and noise was observed.

This study measured the levels of 17 metal elements (Ti, V, Cr, Mn, Fe, Co, Ni, Cu, Zn, As, Se, Sr, Cd, Sb, Ba, Pb, Mo) in the blood of steelworkers. The results showed that the blood metal levels of the study subjects were generally elevated, consistent with previous studies reporting elevated blood metal levels in steel industry workers (26, 27), although specific levels varied. For example, Afridi et al. (28) measured blood Mn and Cu levels of 35.4 μg/L and 910 μg/L, respectively, in steelworkers, which were lower than the results of this study. The blood Mn levels in the steelworkers in this study were higher than those in previously studied populations exposed to Mn (19.82 ± 4.54 μg/L and a median of 30.2 μg/L, respectively) (29, 30). The median blood Cu level of 736.37 μg/L was slightly lower than that of other smelting workers (798–849 μg/L) (31). Blood Se, Sr, and As levels were all lower than those of other lead-exposed workers, suggesting that blood metal levels are significantly influenced by job type and exposure intensity, and that cumulative Mn exposure in the study population may be higher (32).

### 4.1 The association between metal exposure and the risk of hearing loss

The results of the logistic regression model showed that, after adjusting for confounding factors, blood concentrations of Mn, Cu, As, Se, and Sr were significantly positively associated with the risk of hearing loss. After screening using LASSO regression, a total of 10 key metals were identified: titanium, arsenic, molybdenum, zinc, manganese, chromium, selenium, strontium, antimony, and iron. Analysis using the WQS model indicated that the WQS index was significantly associated with the risk of hearing loss, with selenium (Se) and strontium (Sr) being the most heavily weighted metals. The BKMR model further revealed dose-response relationships between strontium (Sr) and manganese (Mn) and hearing loss, and suggested a potential interaction between manganese (Mn) and strontium (Sr).

The primary discrepancy between the WQS and BKMR results was that Se received a high weight in the WQS model, whereas Mn was more prominent in the BKMR model. This difference can be attributed to the distinct assumptions of the two models. WQS imposes directional homogeneity; thus, if Mn exhibits a complex or non-monotonic association with the outcome, or interacts with other metals resulting in inconsistent effect directions, its weight may be underestimated while the contribution of Se may be inflated. In contrast, BKMR flexibly accommodates non-linear and non-additive relationships, enabling the capture of the threshold effect of Mn and the near-exponential pattern of Sr.

In both single-metal and mixed-metal studies, current evidence linking metals to hearing loss is primarily concentrated on lead and cadmium (19, 20, 33, 34). The effects of other metals, such as Co and Sn, on hearing loss remain uncertain based on current research (35). Using NHANES data, Liang and colleagues (33) analyzed the association between combined exposure to multiple metals and hearing loss using the WQS and BKMR models, finding a positive overall association. This suggests that metals such as Cd, Pb, and Sb may exhibit cumulative effects within mixtures, and that potential interactions exist.

Both univariate and multivariate analyses in this study found that Sr and Se were significantly associated with hearing loss among steelworkers. Epidemiological studies generally recognize Se as a trace element with antioxidant properties; by participating in the synthesis of glutathione peroxidase and other selenoproteins, it exerts antioxidant effects that reduce the risk of cardiovascular disease, certain cancers (such as prostate and breast cancer), and metabolic syndrome (36–38). However, similar to the present study, analyses by Chuang et al. (20) and NHANES data (39) also suggest an association between elevated serum selenium concentrations and increased hearing thresholds. It is possible that, at higher exposure levels or when selenoprotein homeostasis is disrupted, selenium may shift from an antioxidant to a pro-oxidant, thereby exacerbating oxidative damage to inner ear hair cells and the auditory nerve (40, 41). This study suggests that selenium may contribute to the process of hearing loss among steelworkers, a population with complex occupational exposures.

Strontium is widely present in the environment and enters the human body primarily through drinking water, food, and occupational exposure. To date, no epidemiological studies or reports have linked blood strontium levels to the risk of hearing loss (42). The results of this study suggest that blood strontium levels have significant weight in a mixed-metal exposure model and exhibit a nonlinear association with hearing loss.

Studies have shown that serum strontium levels may be associated with abnormal bone metabolism, cardiovascular disease, and type 2 diabetes. Potential mechanisms include strontium's competition with calcium ions during the bone mineralization process, interference with calcium signaling pathways and ion homeostasis, and modulation of oxidative stress and inflammatory responses, which may affect the function of relevant tissues. However, the specific mechanisms underlying these effects have not yet been fully elucidated (43–45). The reasons for the association between serum strontium levels and the risk of hearing loss may include: (1) Strontium is distributed in the body similarly to calcium, primarily depositing in bones. It may affect calcium absorption and utilization through competitive metabolism, thereby contributing to hearing loss (42, 46). (2) Sr^2+^ competes with divalent metal ions such as Ca^2+^ during intestinal absorption. Exposure to Sr may alter the absorption efficiency and body distribution of other metal ions, affecting their bioavailability and potential toxic effects in the context of mixed metal exposure (47). In summary, this study suggests a potential association between Sr and hearing loss, but the specific relationship and underlying biological mechanisms require further research to clarify.

Studies have shown that manganese can directly damage the cochlear hair cells and neurons in the spiral ganglion that transmit auditory signals, leading to hearing loss (48). However, in a multivariate regression analysis conducted by Chuang et al. (20) after adjusting for confounding factors such as age, it was primarily lead (Pb) and selenium (Se) concentrations that were significantly associated with hearing thresholds, while manganese (Mn) did not exhibit a significant independent effect.

This study found, through WQS analysis, that other factors potentially influencing hearing loss in mixed exposure include the adverse health effects of arsenic on multiple organ systems under conditions of long-term low-dose exposure. HE et al. (49) found that arsenic may be associated with an increased risk of high-frequency hearing loss (4, 8, 12 kHz) in the general population. However, a recent meta-analysis found no significant association between blood arsenic levels and hearing loss (19). Lead and cadmium are considered potential contributors to hearing loss. Previous studies have shown that elevated blood lead (Pb) and cadmium (Cd) levels are associated with high-frequency hearing decline in adults (50, 51). However, no significant effect was observed in this study, which may be related to abnormal blood Pb and Cd levels in the study population, limited differences in exposure, or the fact that these effects were masked by metals such as Mn and Sr in the context of mixed metal exposure.

### 4.2 Interaction between metals and noise

Occupational noise is considered one of the major risk factors for hearing loss, and environmental or occupational exposure to metals may exacerbate the damaging effects of noise on the auditory system.

This study found an additive interaction between blood manganese (Mn) and strontium (Sr) levels and occupational noise exposure. Possible mechanisms include: (1) Mn induces oxidative stress and participates in the regulation of antioxidant defenses, while Sr, as a group-14 element (the same group as calcium), may disrupt inner ear hair cell function by affecting calcium ion homeostasis and signal transduction processes (52). (2) Noise can also cause oxidative damage to the inner ear and disrupt calcium homeostasis; the combined effects of these three factors may further exacerbate damage to the auditory system (53).

A study by Wu et al. (54) among lead-acid battery manufacturing workers found a synergistic effect on hearing loss when blood lead levels and occupational noise exposure were combined. Hwang et al. (55) found that in a steel mill, elevated blood lead levels below 10 μg/dl may exacerbate noise-induced hearing loss. In contrast, Choi and colleagues' (22) occupational studies emphasized that concurrent exposure to metals and other occupational hazards, such as noise and solvents, may increase the risk of hearing loss, indirectly suggesting that the health risks of multiple exposures should not be overlooked. Furthermore, workers simultaneously exposed to metals and noise in occupational settings face a significantly higher risk of hearing loss than those exposed to noise alone, further supporting the notion that metals may act as a significant synergistic factor in noise-induced hearing loss (56).

### 4.3 Advantages and limitations

This study employed multiple methods to assess the association between exposure to single metals, mixed metals, and hearing loss. First, logistic regression was used to analyze the association between exposure to individual metals and hearing loss among steelworkers. Second, the LASSO regression method was applied to identify key metals; this approach enables variable selection while controlling model complexity, effectively reducing multicollinearity and mitigating the risk of overfitting (56, 57). Finally, the WQS and BKMR models were used to assess the relative contributions of each metal within the mixed-metal exposure and their strength of association with hearing loss. The WQS model quantifies the relative importance of each metal in the overall effect, while the BKMR model flexibly captures the nonlinear effects of individual exposures and interactions between exposures (58–60).

This study has several limitations. First, as an observational research design, with blood metal levels, noise exposure, and hearing loss measured at the same time point, which limits the ability to demonstrate causality. Second, blood metal levels primarily reflect recent exposure and may not fully represent long-term cumulative exposure; moreover, although adjustments were made for various potential confounding factors, the influence of unmeasured or unknown confounders may still exist. Third, the analysis of mixed-metal exposure effects and their interactions relies on statistical model assumptions, which may not fully reflect the actual biological mechanisms. Additionally, occupational noise exposure was assessed qualitatively via a self-reported questionnaire, and metal exposures were dichotomized at the median for interaction analyses, which may affect the stability of the findings and obscure potential dose-response gradients. Finally, the confidence intervals for the interaction estimates were relatively wide, indicating limited precision.

Despite these limitations, this study provides valuable insights into the risk of hearing loss among steelworkers in the context of mixed-metal exposure and lays the groundwork for subsequent longitudinal and mechanistic studies.

## Data Availability

The original contributions presented in the study are included in the article/Supplementary material, further inquiries can be directed to the corresponding authors.
