# Supplementary material for: Effects of mixed blood metals on hearing loss among steel workers: a case-control study
Source: Front Public Health. 2026 Jul 7;14:1863565. doi: 10.3389/fpubh.2026.1863565 (PMC13386853; doi:10.3389/fpubh.2026.1863565)

Supplementary Material

**Supplementary Table 1**

**Interaction between manganese and noise exposure in subgroups**

| **Subgroup** | **Noise exposure** | **Manganese level** | **OR(95%CI)** |
| --- | --- | --- | --- |
| **Speech-frequency**  **Hearing Loss** | No | Low(ref) | 1 |
|  |  | High | 3.05 (0.97, 9.55) |
|  | Yes | Low | 6.33 (1.64, 24.38) |
|  |  | High | 6.94 (1.79, 26.86) |
|  | Multiplicative |  | 0.36 (0.1, 1.28) |
|  | Additive | RERI | -1.44(-19.26,13.27) |
|  |  | AP | -0.21 (-1.48, 0.29) |
|  |  | S | 0.81 (0.4, 1.61) |
| **High-frequency Hearing Loss** | No | Low(ref) | 1 |
|  |  | High | 2.08 (1.02, 4.25) |
|  | Yes | Low | 5.51 (2.08, 14.6) |
|  |  | High | 11.14 (4.09, 30.33) |
|  | Multiplicative |  | 0.97 (0.41, 2.28) |
|  | Additive | RERI | 4.55 (0.58, 20.35) |
|  |  | AP | 0.41 (-0.03, 0.61) |
|  |  | S | 1.81 (1.06, 3.1) |
| **Suspected NIHL** | No | Low(ref) | 1 |
|  |  | High | 1.92 (0.8, 4.61) |
|  | Yes | Low | 4.97 (1.71, 14.45) |
|  |  | High | 6.23 (2.12, 18.31) |
|  | Multiplicative |  | 0.65 (0.24, 1.76) |
|  | Additive | RERI | 0.33 (-7.2, 9.1) |
|  |  | AP | 0.05 (-0.69, 0.41) |
|  |  | S | 1.07 (0.59, 1.92) |

**Supplementary Table 2**

**Interaction between strontium and noise exposure in subgroups**

| **Subgroup** | **Noise exposure** | **Manganese level** | **OR(95%CI)** |
| --- | --- | --- | --- |
| **Speech-frequency**  **Hearing Loss** | No | Low(ref) | 1 |
|  |  | High | 3.6 (1.09, 11.88) |
|  | Yes | Low | 6.45 (1.53, 27.16) |
|  |  | High | 9.54 (2.36, 38.46) |
|  | Multiplicative |  | 0.41 (0.11, 1.52) |
|  | Additive | RERI | 0.49 (-19.6, 23.47) |
|  |  | AP | 0.05 (-0.92, 0.44) |
|  |  | S | 1.06 (0.56, 2.02) |
| **High-frequency Hearing Loss** | No | Low(ref) | 1 |
|  |  | High | 5.33 (2.48, 11.44) |
|  | Yes | Low | 8.46 (2.98, 24.03) |
|  |  | High | 22.09 (7.95, 61.37) |
|  | Multiplicative |  | 0.49 (0.2, 1.19) |
|  | Additive | RERI | 9.3 (-0.02, 42.4) |
|  |  | AP | 0.42 (-0.02, 0.61) |
|  |  | S | 1.79 (1.08, 2.97) |
| **Suspected NIHL** | No | Low(ref) | 1 |
|  |  | High | 2.93 (1.19, 7.26) |
|  | Yes | Low | 5.76 (1.85, 17.91) |
|  |  | High | 9.34 (3.11, 28.07) |
|  | Multiplicative |  | 0.55 (0.2, 1.52) |
|  | Additive | RERI | 1.65 (-7.92, 16.01) |
|  |  | AP | 0.18 (-0.46, 0.46) |
|  |  | S | 1.25 (0.73, 2.14) |

**Supplementary Table 3**

**Model performance of frequency-specific hearing threshold regression analyses**

| **Frequency** | **R²** | **Adjusted R²** | **F** | **P** |
| --- | --- | --- | --- | --- |
| 500 Hz | 0.08 | -0.012 | 0.869 | 0.697 |
| 1000 Hz | 0.083 | -0.009 | 0.900 | 0.645 |
| 2000 Hz | 0.095 | 0.004 | 1.048 | 0.396 |
| 3000 Hz | 0.215 | 0.136 | 2.726 | <0.001 |
| 4000 Hz | 0.211 | 0.132 | 2.673 | <0.001 |
| 6000 Hz | 0.190 | 0.109 | 2.345 | <0.001 |

Note. adjusted for gender, work tenure, noise, high temperature, dust,CO,hypertension,diabetes,dyslipidemia,BMI, education, marital status, alcohol ,smoking,earplug use, mask use

**Supplementary Table 4**

**Frequency-specific associations between blood metal concentrations and hearing thresholds at 3000, 4000, and 6000 Hz**

| **Variable** | **3000hz** | | **4000hz** | | **6000hz** | |
| --- | --- | --- | --- | --- | --- | --- |
|  | **β(95%CI)** | **P** | **β(95%CI)** | **P** | **β(95%CI)** | **P** |
| Ti | -0.023(-0.11,0.063) | 0.6 | -0.026(-0.115,0.062) | 0.556 | -0.023(-0.107,0.061) | 0.592 |
| V | -0.786(-1.691,0.118) | 0.088 | -0.552(-1.476,0.372) | 0.241 | -0.422(-1.296,0.452) | 0.343 |
| Cr | -0.029(-0.066,0.009) | 0.132 | -0.035(-0.073,0.003) | 0.072 | -0.04(-0.076,-0.004) | 0.03 |
| Mn | 0.279(0.095,0.463) | 0.003 | 0.289(0.101,0.477) | 0.003 | 0.289(0.111,0.467) | 0.002 |
| Fe | 3.638E-5(0,0) | 0.005 | 3.724E-5(0,0) | 0.005 | 2.006E-5(0,0) | 0.109 |
| Co | 0.058(-0.094,0.211) | 0.453 | 0.027(-0.129,0.183) | 0.734 | 0.046(-0.102,0.193) | 0.541 |
| Ni | -0.311(-0.529,-0.092) | 0.006 | -0.277(-0.501,-0.053) | 0.015 | -0.304(-0.516,-0.092) | 0.005 |
| Cu | -0.007(-0.018,0.004) | 0.206 | 0.003(-0.009,0.014) | 0.66 | 0.003(-0.007,0.014) | 0.537 |
| Zn | -0.001(-0.001,0) | 0.144 | -0.001(-0.002,0) | 0.085 | -0.001(-0.002,0) | 0.055 |
| As | -0.504(-1.417,0.409) | 0.279 | -0.421(-1.355,0.512) | 0.375 | -0.519(-1.401,0.364) | 0.249 |
| Se | 0.054(-0.025,0.132) | 0.181 | 0.026(-0.054,0.107) | 0.52 | 0.031(-0.045,0.107) | 0.42 |
| Sr | 0.285(0.151,0.419) | <0.001 | 0.263(0.126,0.401) | <0.001 | 0.198(0.068,0.328) | 0.003 |
| Mo | -0.007(-0.03,0.016) | 0.544 | -0.011(-0.035,0.012) | 0.354 | -0.018(-0.04,0.004) | 0.116 |
| Cd | -0.065(-0.463,0.332) | 0.746 | -0.175(-0.581,0.231) | 0.396 | -0.159(-0.543,0.225) | 0.415 |
| Sb | -0.024(-0.083,0.035) | 0.42 | -0.046(-0.107,0.014) | 0.133 | -0.034(-0.091,0.023) | 0.246 |
| Ba | -0.016(-0.039,0.007) | 0.162 | -0.013(-0.036,0.01) | 0.26 | -0.011(-0.032,0.011) | 0.34 |
| Pb | 0.092(-0.041,0.226) | 0.174 | 0.207(0.07,0.343) | 0.003 | 0.229(0.1,0.358) | 0.001 |

Note. adjusted for gender, work tenure, noise, high temperature, dust,CO,hypertension,diabetes,dyslipidemia,BMI, education, marital status, alcohol ,smoking,earplug use, mask use

**Supplementary Figure 1**

**The average audiogram of the hearing loss cases**


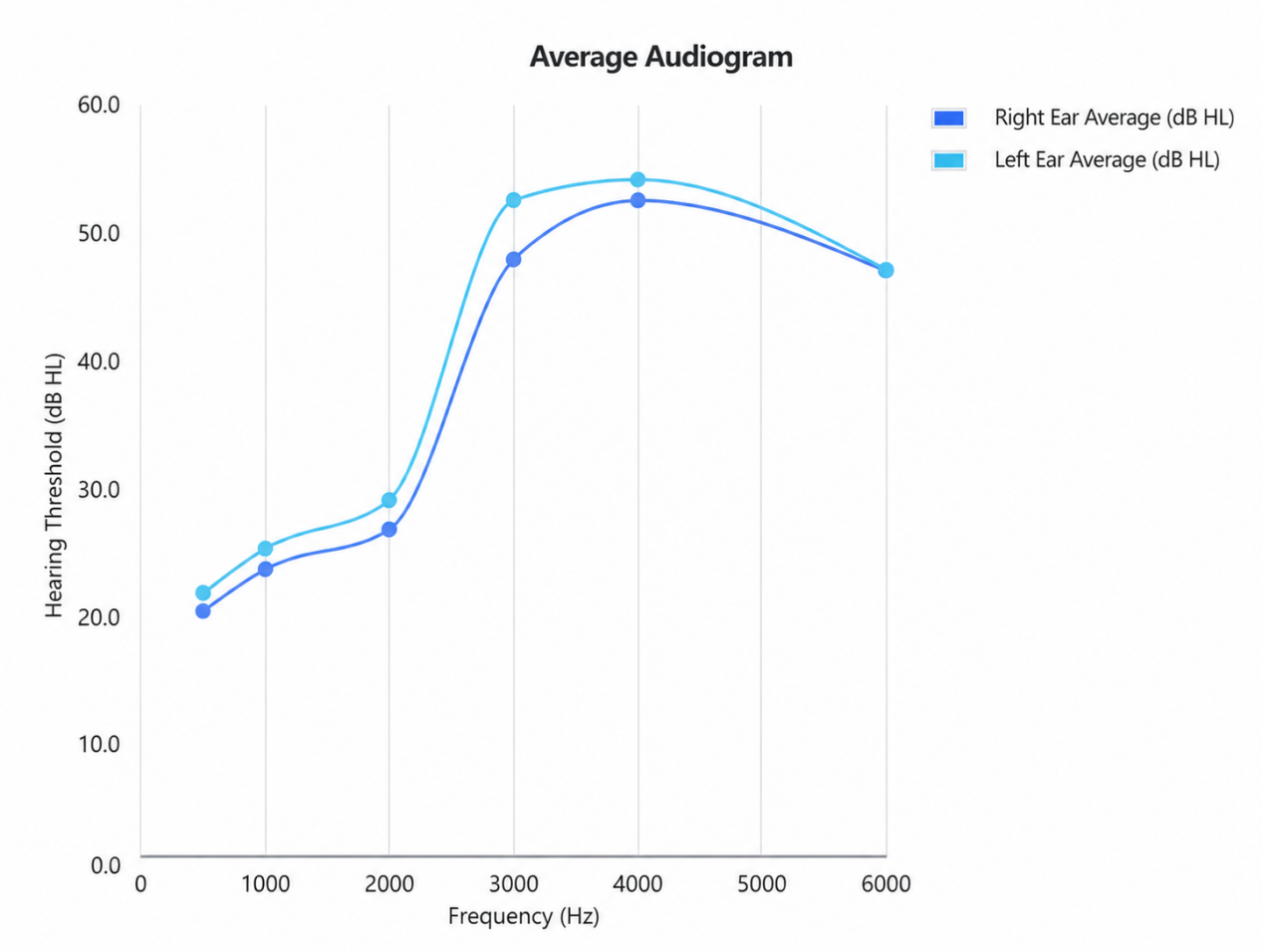

Supplement: Supplementary file 1 [file Table_1.docx]
